# Ethyl 2-[(*Z*)-3-chloro­benzyl­idene]-7-methyl-3-oxo-5-phenyl-2,3-dihydro-5*H*-1,3-thia­zolo[3,2-*a*]pyrimidine-6-carboxyl­ate

**DOI:** 10.1107/S1600536808007356

**Published:** 2008-03-29

**Authors:** Mukesh M. Jotani, Bharat B. Baldaniya

**Affiliations:** a Bhavan’s R. A. College of Science, Ahmedabd, Gujarat 380 001, India; b M. G. Science Institute, Navrangpura, Ahmedabad, Gujarat 380 009, India

## Abstract

In the title compound, C_23_H_19_ClN_2_O_3_S, the central pyrimidine ring is significantly puckered, assuming almost a screw boat conformation. In addition to the usual inter­molecular C—H⋯O hydrogen bonding, short intra­molecular C—H⋯S contacts and π–π stacking inter­actions [centroid–centroid distance = 3.762 (2) Å] contribute to the crystal packing.

## Related literature

For the crystal structures of similar compounds, see: Jotani & Baldaniya (2006 ▶, 2007 ▶); Sridhar *et al.*, (2006 ▶); Fischer *et al.* (2007 ▶). For the biological activities, see: Kappe (2000 ▶); Rovnyak *et al.* (1995 ▶); Monks *et al.* (1991 ▶); Winter *et al.* (1962 ▶). For related literature, see: Allen, (2002 ▶); Bernstein *et al.* (1995 ▶); Cremer & Pople, (1975 ▶).
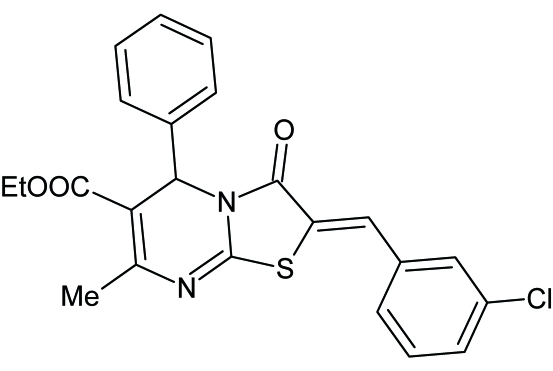


## Experimental

### 

#### Crystal data

C_23_H_19_ClN_2_O_3_S
*M* *_r_* = 438.92
Triclinic, 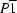

*a* = 8.2650 (3) Å
*b* = 10.3291 (4) Å
*c* = 13.5655 (5) Å
α = 94.129 (2)°
β = 100.837 (2)°
γ = 111.812 (2)°
*V* = 1043.15 (7) Å^3^
*Z* = 2
Mo *K*α radiation
μ = 0.31 mm^−1^
*T* = 293 (2) K
0.47 × 0.35 × 0.2 mm

#### Data collection

Bruker Kappa APEXII CCD diffractometer
Absorption correction: multi-scan (*SADABS*; Bruker, 2004 ▶) *T*_min_ = 0.867, *T*_max_ = 0.936
18826 measured reflections
3677 independent reflections
3111 reflections with *I* > 2σ(*I*)
*R*_int_ = 0.024

#### Refinement

*R*[*F* ^2^ > 2σ(*F* ^2^)] = 0.041
*wR*(*F* ^2^) = 0.124
*S* = 1.06
3677 reflections
273 parameters
H-atom parameters constrained
Δρ_max_ = 0.22 e Å^−3^
Δρ_min_ = −0.25 e Å^−3^

### 

Data collection: *APEX2* (Bruker, 2004 ▶); cell refinement: *APEX2* and *SAINT* (Bruker, 2004 ▶); data reduction: *SAINT* and *XPREP* (Bruker, 2004 ▶); program(s) used to solve structure: *SIR97* (Altomare *et al.*, 1999 ▶); program(s) used to refine structure: *SHELXL97* (Sheldrick, 2008 ▶); molecular graphics: *ORTEP-3* (Farrugia, 1997 ▶) and *PLATON* (Spek, 2003 ▶); software used to prepare material for publication: *SHELXL97* and *PLATON*.

## Supplementary Material

Crystal structure: contains datablocks global, I. DOI: 10.1107/S1600536808007356/rk2081sup1.cif

Structure factors: contains datablocks I. DOI: 10.1107/S1600536808007356/rk2081Isup2.hkl

Additional supplementary materials:  crystallographic information; 3D view; checkCIF report

## Figures and Tables

**Table 1:** Hydrogen-bond geometry (Å, °)

*D*—H⋯*A*	*D*—H	H⋯*A*	*D*⋯*A*	*D*—H⋯*A*
C23—H23⋯O1^i^	0.93	2.49	3.287 (3)	143
C19—H19⋯S1	0.93	2.50	3.210 (3)	133

Symmetry code: (i) 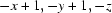.

**Fig. 1.**
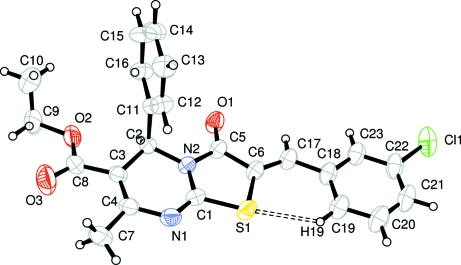
The molecular structure of I with the atom numbering scheme. Displacement ellipsoids are drawn at 50% probability level. H atoms are presented as a small spheres of arbitrary radius. The C—H···S intramolecular hydrogen bond is indicated by dashed line.

**Fig. 2.**
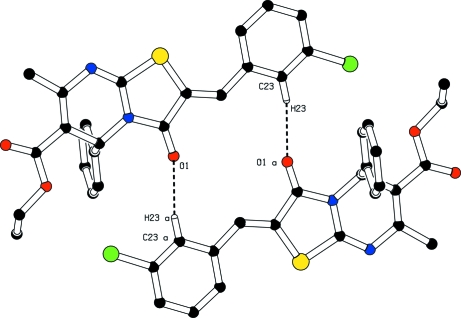
PLATON (Spek, 2003) plot of I showing C—H···O intermolecular interactions and forming R22(14) graph–set motif as dashed lines. H atoms not involved in hydrogen bonding have been omitted. Symmetry code (i): -x+1, -y+1, -z.

**Fig. 3.**
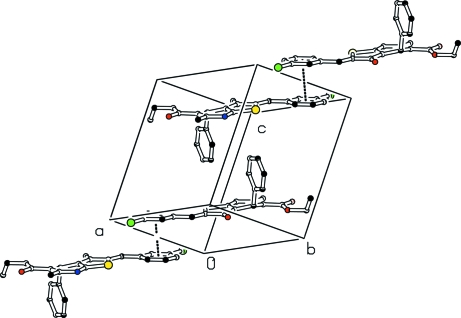
A view of the π–π stacking interaction (dashed line) in the crystal structure of I. H atoms have been omitted for clarity.

## supplementary crystallographic information

### Comment

The remarkable biological activties of fused pyrimidines such as antiviral, antitumor, anticancer, antiinflammatory, antihypertensive *etc*. (Kappe, 2000; Rovnyak *et al.*, 1995) bring this class of heterocyclic compounds of significant pharmacological interest. The title thiazolo [3,2-*a*] pyrimidine compound, (I), possesses anticancer and antiinflammatory activity. The anticancer drug screen is carried out using a diverse panel of cultured human tumor cell lines (Monks *et al.*, 1991). The anti-inflammatory activity is determined by inhibition in the Carageena-induced rat paw edema method (Winter *et al.*, 1962). In view of these properties and to study the effect of chlorine substituent at different positions of benzene ring on crystal packing, the crystal structure of I has been determined.

On Fig. 1 is shown the molecular structure of I with the atom numbering scheme. The pyrimidine ring adopts almost a screw boat conformation as indicated by the ring puckering parameters [q2 = 0.175 (2)Å, q3 = 0.077 (2)Å, θ = 66.2 (6)° and φ = 173.1 (7)°; Cremer & Pople, 1975]. The idealized values for the screw boat conformation are: θ = 67.5° and φ = (60k + 30)°, where k is an integer. The asymmetry parameters also support this information. All bond lengths and angles in the pyrimidine ring have normal values. The fused thiazole ring has usual geometry as observed in other fused thiazolopyrimidine compounds (Jotani & Baldaniya, 2006, 2007; Sridhar *et al.*, 2006). The thiazole ring makes dihedral angles of 89.06 (11) and 6.56 (10)° with the benzene rings C11–C16 and C18–C23, respectively. The short C9–C10 bond distance is a consequence of slight liberation of the ethoxy group. The ethoxy group is in all–*trans* conformation as observed from the torsional angles C3–C8–O2–C9 and C8–O2–C9–C10 of -176.0 (2) and 117.2 (3)° respectively.

The crystal structure of I is also have an intermolecular C—H···O interaction (Fig. 2 and Table) similar to earlier reported *ortho*- and *para*-substituted compounds (Jotani & Baldaniya, 2006, 2007). In the structure a carbon C23 atom of C18–C23 benzene ring participates in the C—H···O interaction that forms *R*_2_^2^(14) graph–set motif (Bernstein *et al.*, 1995), where as in *ortho*- and *para*-substituted chlorine derivatives, the interaction is due to C14 atom of C11–C16 benzene ring. This may be the different symmetry of the crystal system as the title compound I crystallizes in triclinic system where as the previous two structures were crystallized in monoclinic space group. A *PLATON* analysis (Spek, 2003) of I indicated that a weak intramolecular C—H···S hydrogen bond (Fig. 1 and Table) forms a pseudo-six-membered ring of S(6) graph-set motif (Bernstein *et al.*, 1995) also help to consolidate the crystal packing. There is a comparatively weak π–π stacking interaction between the C18–C23 benzene rings with symmetry codes: (*x*, *y*, *z*) and (1-*x*, 1-*y*, -*z*); their centroids are separated by 3.762 (2)Å and the rings have a slippage of 1.523Å (Fig. 3).

### Experimental

A mixture of ethyl 6-methyl-4-phenyl-2-thioxo-1,2,3,4-tetrahydropyrimidine-5-carboxylate (0.01 mol), chloroacetic acid (0.01 mol), fused sodium acetate (6 g) in glacial acetic acid (25 ml), acetic anhydride (10 ml) and 3-chlorobenzaldehyde (0.01 mol) was refluxed for 3 h. The reaction mixture was cooled and poured into cold water. The resulting solid was collected and crystallized from methanol to obtain the final product (82% yield, mp 444 K). The compound was recrystallized by slow evaporation of an ethanol solution, yielding yellow, Platlike single crystals suitable for X–ray diffraction.

### Refinement

H atoms were placed in idealized positions (C—H = 0.93–0.98Å) and constrained to ride on their parent atoms with *U*_iso_(H) = 1.5*U*_eq_(C) for methyl H atoms and 1.2*U*_eq_(C) for other H atoms.

### Crystal data

**Table:**

C_23_H_19_ClN_2_O_3_S	*Z* = 2
*M**_r_* = 438.92	*F*_000_ = 456
Triclinic, *P*1	*D*_x_ = 1.397 Mg m^−^^3^
Hall symbol: -P 1	Mo *K*α radiation λ = 0.71073 Å
*a* = 8.2650 (3) Å	Cell parameters from 3579 reflections
*b* = 10.3291 (4) Å	θ = 2.4–25.0º
*c* = 13.5655 (5) Å	µ = 0.31 mm^−^^1^
α = 94.129 (2)º	*T* = 293 (2) K
β = 100.837 (2)º	Plate, yellow
γ = 111.812 (2)º	0.47 × 0.35 × 0.2 mm
*V* = 1043.15 (7) Å^3^	

### Data collection

**Table:**

Bruker KappaAPEXII CCD diffractometer	3677 independent reflections
Radiation source: Fine–focus sealed tube	3111 reflections with *I* > 2σ(*I*)
Monochromator: Graphite	*R*_int_ = 0.024
*T* = 293(2) K	θ_max_ = 25.0º
ω and φ scans	θ_min_ = 2.2º
Absorption correction: multi-scan(SADABS; Bruker, 2004)	*h* = −9→9
*T*_min_ = 0.867, *T*_max_ = 0.936	*k* = −12→12
18826 measured reflections	*l* = −16→16

### Refinement

**Table:**

Refinement on *F*^2^	Secondary atom site location: Difmap
Least-squares matrix: Full	Hydrogen site location: Geom
*R*[*F*^2^ > 2σ(*F*^2^)] = 0.041	H-atom parameters constrained
*wR*(*F*^2^) = 0.124	*w* = 1/[σ^2^(*F*_o_^2^) + (0.0643*P*)^2^ + 0.3244*P*] where *P* = (*F*_o_^2^ + 2*F*_c_^2^)/3
*S* = 1.06	(Δ/σ)_max_ = 0.007
3677 reflections	Δρ_max_ = 0.22 e Å^−^^3^
273 parameters	Δρ_min_ = −0.25 e Å^−^^3^
Primary atom site location: Direct	Extinction correction: None

### Special details

**Table:**

Geometry. All s.u.'s (except the s.u. in the dihedral angle between two l.s. planes) are estimated using the full covariance matrix. The cell s.u.'s are taken into account individually in the estimation of s.u.'s in distances, angles and torsion angles; correlations between s.u.'s in cell parameters are only used when they are defined by crystal symmetry. An approximate (isotropic) treatment of cell s.u.'s is used for estimating s.u.'s involving l.s. planes.
Refinement. Refinement of *F*^2^ against ALL reflections. The weighted *R*–factor w*R* and goodness of fit *S* are based on *F*^2^, conventional *R*–factors *R* are based on *F*, with *F* set to zero for negative *F*^2^. The threshold expression of *F*^2^ > 2σ(*F*^2^) is used only for calculating *R*–factors(gt) etc. and is not relevant to the choice of reflections for refinement. *R*–factors based on *F*^2^ are statistically about twice as large as those based on *F*, and *R*–factors based on ALL data will be even larger.

### Fractional atomic coordinates and isotropic or equivalent isotropic displacement parameters (Å^2^)

**Table:**

	*x*	*y*	*z*	*U*_iso_*/*U*_eq_	
S1	0.30130 (8)	0.19021 (5)	0.22916 (4)	0.06087 (19)	
Cl1	1.00143 (9)	0.23402 (9)	−0.04622 (5)	0.0932 (3)	
N1	0.0766 (2)	0.24166 (16)	0.32599 (13)	0.0579 (4)	
N2	0.2084 (2)	0.40286 (15)	0.22081 (11)	0.0471 (4)	
O1	0.3663 (2)	0.52920 (15)	0.11435 (11)	0.0657 (4)	
O2	−0.0956 (2)	0.62159 (17)	0.26634 (16)	0.0856 (6)	
O3	−0.2281 (3)	0.4916 (2)	0.37203 (18)	0.1084 (7)	
C1	0.1793 (3)	0.28444 (18)	0.26598 (14)	0.0497 (4)	
C2	0.1416 (2)	0.50926 (18)	0.25273 (14)	0.0450 (4)	
H2	0.0929	0.5415	0.1923	0.054*	
C3	−0.0077 (2)	0.44015 (19)	0.30586 (14)	0.0503 (4)	
C4	−0.0276 (3)	0.3192 (2)	0.34291 (15)	0.0545 (5)	
C5	0.3266 (3)	0.4282 (2)	0.15758 (14)	0.0502 (4)	
C6	0.3964 (3)	0.3146 (2)	0.15427 (14)	0.0525 (5)	
C7	−0.1590 (3)	0.2496 (3)	0.4043 (2)	0.0770 (7)	
H7A	−0.1239	0.3051	0.4704	0.115*	
H7B	−0.1614	0.1571	0.4111	0.115*	
H7C	−0.2760	0.2420	0.3707	0.115*	
C8	−0.1222 (3)	0.5170 (2)	0.32026 (17)	0.0598 (5)	
C9	−0.1899 (4)	0.7149 (3)	0.2770 (3)	0.1048 (11)	
H9A	−0.2618	0.7134	0.2112	0.126*	
H9B	−0.2695	0.6817	0.3222	0.126*	
C10	−0.0642 (5)	0.8562 (3)	0.3169 (3)	0.1099 (10)	
H10A	−0.1276	0.9165	0.3232	0.165*	
H10B	0.0139	0.8890	0.2718	0.165*	
H10C	0.0054	0.8577	0.3825	0.165*	
C11	0.2941 (2)	0.63460 (17)	0.32116 (13)	0.0427 (4)	
C12	0.3852 (3)	0.6173 (2)	0.41182 (15)	0.0600 (5)	
H12	0.3526	0.5284	0.4313	0.072*	
C13	0.5242 (3)	0.7309 (3)	0.47383 (17)	0.0704 (6)	
H13	0.5879	0.7179	0.5339	0.085*	
C14	0.5688 (3)	0.8626 (2)	0.44719 (19)	0.0709 (6)	
H14	0.6613	0.9397	0.4895	0.085*	
C15	0.4773 (4)	0.8804 (2)	0.3587 (2)	0.0813 (7)	
H15	0.5068	0.9702	0.3408	0.098*	
C16	0.3416 (3)	0.7672 (2)	0.29520 (17)	0.0640 (5)	
H16	0.2815	0.7806	0.2342	0.077*	
C17	0.5194 (3)	0.3207 (2)	0.10123 (15)	0.0572 (5)	
H17	0.5494	0.3961	0.0651	0.069*	
C18	0.6139 (3)	0.2279 (2)	0.09120 (14)	0.0573 (5)	
C19	0.5795 (3)	0.1033 (3)	0.13257 (17)	0.0684 (6)	
H19	0.4902	0.0744	0.1686	0.082*	
C20	0.6773 (4)	0.0225 (3)	0.12037 (19)	0.0801 (7)	
H20	0.6543	−0.0597	0.1491	0.096*	
C21	0.8079 (4)	0.0619 (3)	0.06648 (18)	0.0763 (7)	
H21	0.8734	0.0072	0.0584	0.092*	
C22	0.8402 (3)	0.1837 (3)	0.02458 (16)	0.0677 (6)	
C23	0.7458 (3)	0.2672 (2)	0.03618 (15)	0.0608 (5)	
H23	0.7704	0.3494	0.0073	0.073*	

### Atomic displacement parameters (Å^2^)

**Table:**

	*U*^11^	*U*^22^	*U*^33^	*U*^12^	*U*^13^	*U*^23^
S1	0.0788 (4)	0.0511 (3)	0.0654 (3)	0.0353 (3)	0.0227 (3)	0.0169 (2)
Cl1	0.0854 (5)	0.1389 (7)	0.0814 (4)	0.0718 (5)	0.0248 (3)	0.0110 (4)
N1	0.0634 (10)	0.0453 (9)	0.0625 (10)	0.0157 (8)	0.0188 (8)	0.0127 (8)
N2	0.0545 (9)	0.0406 (8)	0.0479 (8)	0.0202 (7)	0.0130 (7)	0.0064 (6)
O1	0.0892 (10)	0.0631 (9)	0.0723 (9)	0.0459 (8)	0.0416 (8)	0.0308 (7)
O2	0.0723 (10)	0.0714 (10)	0.1448 (17)	0.0428 (8)	0.0596 (11)	0.0413 (11)
O3	0.1136 (15)	0.1288 (17)	0.1383 (18)	0.0757 (14)	0.0846 (14)	0.0613 (14)
C1	0.0559 (11)	0.0402 (9)	0.0495 (10)	0.0167 (8)	0.0089 (8)	0.0058 (8)
C2	0.0469 (9)	0.0414 (9)	0.0494 (10)	0.0191 (8)	0.0133 (8)	0.0083 (7)
C3	0.0432 (9)	0.0455 (10)	0.0538 (11)	0.0090 (8)	0.0112 (8)	0.0032 (8)
C4	0.0492 (10)	0.0471 (10)	0.0570 (11)	0.0080 (8)	0.0134 (8)	0.0041 (8)
C5	0.0608 (11)	0.0499 (10)	0.0464 (10)	0.0273 (9)	0.0151 (8)	0.0099 (8)
C6	0.0641 (11)	0.0518 (10)	0.0480 (10)	0.0311 (9)	0.0104 (9)	0.0082 (8)
C7	0.0740 (15)	0.0635 (14)	0.0905 (17)	0.0127 (11)	0.0392 (13)	0.0200 (12)
C8	0.0462 (10)	0.0597 (12)	0.0715 (13)	0.0167 (9)	0.0195 (9)	0.0058 (10)
C9	0.0828 (18)	0.088 (2)	0.180 (3)	0.0567 (16)	0.061 (2)	0.038 (2)
C10	0.112 (2)	0.086 (2)	0.150 (3)	0.0586 (19)	0.037 (2)	0.0105 (19)
C11	0.0403 (8)	0.0421 (9)	0.0494 (10)	0.0171 (7)	0.0170 (7)	0.0064 (7)
C12	0.0666 (12)	0.0487 (11)	0.0581 (12)	0.0187 (9)	0.0066 (10)	0.0087 (9)
C13	0.0690 (14)	0.0758 (15)	0.0564 (12)	0.0245 (12)	0.0022 (10)	0.0014 (11)
C14	0.0588 (12)	0.0558 (13)	0.0766 (15)	0.0026 (10)	0.0148 (11)	−0.0080 (11)
C15	0.0806 (16)	0.0443 (12)	0.0954 (18)	0.0020 (11)	0.0115 (14)	0.0139 (12)
C16	0.0650 (13)	0.0484 (11)	0.0705 (13)	0.0143 (10)	0.0104 (10)	0.0174 (10)
C17	0.0714 (13)	0.0622 (12)	0.0508 (11)	0.0388 (10)	0.0160 (9)	0.0127 (9)
C18	0.0697 (12)	0.0682 (13)	0.0441 (10)	0.0422 (11)	0.0064 (9)	0.0055 (9)
C19	0.0897 (16)	0.0763 (14)	0.0588 (12)	0.0526 (13)	0.0189 (11)	0.0164 (11)
C20	0.109 (2)	0.0881 (17)	0.0676 (14)	0.0677 (16)	0.0141 (14)	0.0181 (12)
C21	0.0910 (17)	0.0959 (18)	0.0632 (13)	0.0684 (15)	0.0039 (12)	0.0043 (13)
C22	0.0688 (13)	0.0962 (17)	0.0486 (11)	0.0526 (13)	0.0013 (9)	−0.0021 (11)
C23	0.0686 (13)	0.0753 (14)	0.0477 (11)	0.0430 (11)	0.0056 (9)	0.0059 (9)

### Geometric parameters (Å, °)

**Table:**

S1—C6	1.743 (2)	C10—H10A	0.9600
S1—C1	1.750 (2)	C10—H10B	0.9600
Cl1—C22	1.734 (3)	C10—H10C	0.9600
N1—C1	1.269 (2)	C11—C16	1.370 (3)
N1—C4	1.413 (3)	C11—C12	1.376 (3)
N2—C1	1.369 (2)	C12—C13	1.378 (3)
N2—C5	1.384 (2)	C12—H12	0.9300
N2—C2	1.474 (2)	C13—C14	1.366 (3)
O1—C5	1.202 (2)	C13—H13	0.9300
O2—C8	1.322 (3)	C14—C15	1.357 (4)
O2—C9	1.461 (3)	C14—H14	0.9300
O3—C8	1.190 (3)	C15—C16	1.372 (3)
C2—C3	1.518 (3)	C15—H15	0.9300
C2—C11	1.517 (2)	C16—H16	0.9300
C2—H2	0.9800	C17—C18	1.458 (3)
C3—C4	1.345 (3)	C17—H17	0.9300
C3—C8	1.473 (3)	C18—C23	1.390 (3)
C4—C7	1.495 (3)	C18—C19	1.395 (3)
C5—C6	1.489 (3)	C19—C20	1.382 (3)
C6—C17	1.338 (3)	C19—H19	0.9300
C7—H7A	0.9600	C20—C21	1.370 (4)
C7—H7B	0.9600	C20—H20	0.9300
C7—H7C	0.9600	C21—C22	1.374 (4)
C9—C10	1.432 (4)	C21—H21	0.9300
C9—H9A	0.9700	C22—C23	1.380 (3)
C9—H9B	0.9700	C23—H23	0.9300
			
C6—S1—C1	91.75 (9)	H10A—C10—H10B	109.5
C1—N1—C4	116.63 (16)	C9—C10—H10C	109.5
C1—N2—C5	116.72 (15)	H10A—C10—H10C	109.5
C1—N2—C2	120.56 (15)	H10B—C10—H10C	109.5
C5—N2—C2	121.87 (14)	C16—C11—C12	118.73 (17)
C8—O2—C9	118.6 (2)	C16—C11—C2	120.90 (17)
N1—C1—N2	126.94 (18)	C12—C11—C2	120.36 (16)
N1—C1—S1	121.68 (15)	C11—C12—C13	120.4 (2)
N2—C1—S1	111.37 (14)	C11—C12—H12	119.8
N2—C2—C3	108.40 (14)	C13—C12—H12	119.8
N2—C2—C11	109.88 (14)	C14—C13—C12	120.0 (2)
C3—C2—C11	111.85 (14)	C14—C13—H13	120.0
N2—C2—H2	108.9	C12—C13—H13	120.0
C3—C2—H2	108.9	C15—C14—C13	119.6 (2)
C11—C2—H2	108.9	C15—C14—H14	120.2
C4—C3—C8	121.80 (18)	C13—C14—H14	120.2
C4—C3—C2	121.61 (17)	C14—C15—C16	120.7 (2)
C8—C3—C2	116.49 (17)	C14—C15—H15	119.7
C3—C4—N1	122.24 (17)	C16—C15—H15	119.7
C3—C4—C7	125.8 (2)	C11—C16—C15	120.4 (2)
N1—C4—C7	111.97 (18)	C11—C16—H16	119.8
O1—C5—N2	123.64 (17)	C15—C16—H16	119.8
O1—C5—C6	126.67 (18)	C6—C17—C18	130.16 (19)
N2—C5—C6	109.69 (16)	C6—C17—H17	114.9
C17—C6—C5	120.14 (18)	C18—C17—H17	114.9
C17—C6—S1	129.37 (15)	C23—C18—C19	118.59 (19)
C5—C6—S1	110.45 (14)	C23—C18—C17	117.16 (19)
C4—C7—H7A	109.5	C19—C18—C17	124.2 (2)
C4—C7—H7B	109.5	C20—C19—C18	120.3 (2)
H7A—C7—H7B	109.5	C20—C19—H19	119.8
C4—C7—H7C	109.5	C18—C19—H19	119.8
H7A—C7—H7C	109.5	C21—C20—C19	120.9 (2)
H7B—C7—H7C	109.5	C21—C20—H20	119.6
O3—C8—O2	122.0 (2)	C19—C20—H20	119.6
O3—C8—C3	126.6 (2)	C20—C21—C22	118.8 (2)
O2—C8—C3	111.39 (17)	C20—C21—H21	120.6
C10—C9—O2	110.2 (2)	C22—C21—H21	120.6
C10—C9—H9A	109.6	C21—C22—C23	121.7 (2)
O2—C9—H9A	109.6	C21—C22—Cl1	119.69 (17)
C10—C9—H9B	109.6	C23—C22—Cl1	118.6 (2)
O2—C9—H9B	109.6	C22—C23—C18	119.7 (2)
H9A—C9—H9B	108.1	C22—C23—H23	120.2
C9—C10—H10A	109.5	C18—C23—H23	120.2
C9—C10—H10B	109.5		
			
C4—N1—C1—N2	4.5 (3)	C9—O2—C8—C3	−176.0 (2)
C4—N1—C1—S1	−174.31 (14)	C4—C3—C8—O3	7.8 (4)
C5—N2—C1—N1	−179.82 (18)	C2—C3—C8—O3	−168.5 (2)
C2—N2—C1—N1	10.6 (3)	C4—C3—C8—O2	−171.50 (19)
C5—N2—C1—S1	−0.9 (2)	C2—C3—C8—O2	12.2 (3)
C2—N2—C1—S1	−170.54 (12)	C8—O2—C9—C10	117.2 (3)
C6—S1—C1—N1	−179.77 (17)	N2—C2—C11—C16	120.27 (19)
C6—S1—C1—N2	1.29 (14)	C3—C2—C11—C16	−119.3 (2)
C1—N2—C2—C3	−20.5 (2)	N2—C2—C11—C12	−61.0 (2)
C5—N2—C2—C3	170.42 (15)	C3—C2—C11—C12	59.4 (2)
C1—N2—C2—C11	101.98 (18)	C16—C11—C12—C13	−1.6 (3)
C5—N2—C2—C11	−67.1 (2)	C2—C11—C12—C13	179.69 (19)
N2—C2—C3—C4	18.8 (2)	C11—C12—C13—C14	2.2 (3)
C11—C2—C3—C4	−102.5 (2)	C12—C13—C14—C15	−1.1 (4)
N2—C2—C3—C8	−164.91 (16)	C13—C14—C15—C16	−0.6 (4)
C11—C2—C3—C8	73.8 (2)	C12—C11—C16—C15	−0.1 (3)
C8—C3—C4—N1	177.36 (17)	C2—C11—C16—C15	178.6 (2)
C2—C3—C4—N1	−6.5 (3)	C14—C15—C16—C11	1.2 (4)
C8—C3—C4—C7	−1.9 (3)	C5—C6—C17—C18	176.23 (19)
C2—C3—C4—C7	174.21 (19)	S1—C6—C17—C18	−1.3 (4)
C1—N1—C4—C3	−6.4 (3)	C6—C17—C18—C23	−175.3 (2)
C1—N1—C4—C7	173.01 (18)	C6—C17—C18—C19	5.1 (4)
C1—N2—C5—O1	−178.87 (18)	C23—C18—C19—C20	1.2 (3)
C2—N2—C5—O1	−9.4 (3)	C17—C18—C19—C20	−179.2 (2)
C1—N2—C5—C6	0.0 (2)	C18—C19—C20—C21	−0.9 (4)
C2—N2—C5—C6	169.41 (15)	C19—C20—C21—C22	0.0 (4)
O1—C5—C6—C17	1.8 (3)	C20—C21—C22—C23	0.5 (3)
N2—C5—C6—C17	−176.99 (17)	C20—C21—C22—Cl1	−178.66 (18)
O1—C5—C6—S1	179.79 (17)	C21—C22—C23—C18	−0.2 (3)
N2—C5—C6—S1	1.0 (2)	Cl1—C22—C23—C18	178.96 (15)
C1—S1—C6—C17	176.5 (2)	C19—C18—C23—C22	−0.6 (3)
C1—S1—C6—C5	−1.29 (14)	C17—C18—C23—C22	179.71 (18)
C9—O2—C8—O3	4.7 (4)		

### Hydrogen-bond geometry (Å, °)

**Table:**

*D*—H···*A*	*D*—H	H···*A*	*D*···*A*	*D*—H···*A*
C23—H23···O1^i^	0.93	2.49	3.287 (3)	143
C19—H19···S1	0.93	2.50	3.210 (3)	133

Symmetry codes: (i) −*x*+1, −*y*+1, −*z*.
